# Differential patterns of age‐related cortical and subcortical functional connectivity in 6‐to‐10 year old children: A connectome‐wide association study

**DOI:** 10.1002/brb3.1031

**Published:** 2018-06-30

**Authors:** Carolyn D. Langen, Ryan Muetzel, Laura Blanken, Aad van der Lugt, Henning Tiemeier, Frank Verhulst, Wiro J. Niessen, Tonya White

**Affiliations:** ^1^ Department of Radiology and Nuclear Medicine Erasmus MC Rotterdam The Netherlands; ^2^ Department of Medical Informatics Erasmus MC Rotterdam The Netherlands; ^3^ Department of Child and Adolescent Psychiatry Erasmus MC‐Sophia Children's Hospital Rotterdam The Netherlands; ^4^ The Generation R Study Group Erasmus MC Rotterdam The Netherlands; ^5^ Department of Epidemiology Erasmus MC Rotterdam The Netherlands; ^6^ Imaging Physics Faculty of Applied Sciences Delft University of Technology Delft The Netherlands

**Keywords:** brain development, brain networks, children, connectome, functional MRI, resting‐state

## Abstract

**Introduction:**

Typical brain development is characterized by specific patterns of maturation of functional networks. Cortico‐cortical connectivity generally increases, whereas subcortico‐cortical connections often decrease. Little is known about connectivity changes amongst different subcortical regions in typical development.

**Methods:**

This study examined age‐ and gender‐related differences in functional connectivity between and within cortical and subcortical regions using two different approaches. The participants included 411 six‐ to ten‐year‐old typically developing children sampled from the population‐based Generation R study. Functional connectomes were defined in native space using regions of interest from subject‐specific FreeSurfer segmentations. Connections were defined as: (a) the correlation between regional mean time‐series; and (b) the focal maximum of voxel‐wise correlations within FreeSurfer regions. The association of age and gender with each functional connection was determined using linear regression. The preprocessing included the exclusion of children with excessive head motion and scrubbing to reduce the influence of minor head motion during scanning.

**Results:**

Cortico‐cortical associations echoed previous findings that connectivity shifts from short to long‐range with age. Subcortico‐cortical associations with age were primarily negative in the focal network approach but were both positive and negative in the mean time‐series network approach. Between subcortical regions, age‐related associations were negative in both network approaches. Few connections had significant associations with gender.

**Conclusions:**

The present study replicates previously reported age‐related patterns of connectivity in a relatively narrow age‐range of children. In addition, we extended these findings by demonstrating decreased connectivity within the subcortex with increasing age. Lastly, we show the utility of a more focal approach that challenges the spatial assumptions made by the traditional mean time series approach.

## 1 INTRODUCTION

Understanding typical brain development is critical to understanding the mechanisms behind neuropsychiatric disorders. Mental health in adulthood is highly dependent on brain development beginning in the womb and continuing throughout adolescence and into adulthood. One theory is that the neurobiological underpinnings of mental illnesses are largely driven by atypical brain connectivity originating in childhood (Di Martino et al., 2014; Menon, 2013). Through an understanding of typical connectivity, we can identify aberrant patterns associated with neuropsychiatric disorders.

Functional connectivity changes dramatically in the early years of life. In infancy, the brain's short‐range connections are dominant (Gao et al., 2011; Di Martino et al., 2014). Throughout childhood and adolescence, functional connectivity becomes increasingly distributed, with long‐range connections becoming stronger and short‐range connectivity decreasing (Fair et al., 2009; Di Martino et al., 2014; Rubia, 2013). Furthermore, graph theory studies have also demonstrated that while topological features of brain connectivity are mature by age eight, the hierarchical and modularity of global brain networks continues to mature into adulthood (Menon, 2013).

Functional connectivity between subcortical and cortical regions has been shown to decrease with age in children (Cerliani et al., 2015; Greene et al., 2014; Sato et al., 2015; Supekar, Musen, & Menon, 2009). However, other studies have found the opposite pattern (Sato et al., 2015; Solé‐padullés et al., 2015). Age‐related differences in functional connectivity between subcortical and cortical regions are accompanied by stronger cortico‐cortical connectivity in older children (Supekar et al., 2009). There have been few studies examining the role of connections between different subcortical brain structures in children. Gaining a better understanding of the age‐related development of subcortical functional connectivity provides an important baseline for the study of childhood psychopathology.

Development of brain connectivity is increasingly being studied using whole‐brain connectomes derived from resting‐state functional MRI (rs‐fMRI; Di Martino et al., 2014; Rubia, 2013). Connectomes represent brain connectivity between pairs of grey matter ROI's (Bullmore & Sporns, 2009; Rubinov & Sporns, 2010). Since connectome approaches evaluate networks within the entire brain, they are well suited to evaluate the major changes taking place in typical neurodevelopment.

In this study, we utilized two connectome approaches to evaluate age and gender associations in a large group of school age children across the functional connectome. First, we used the correlation of the mean time series for brain regions involved in a given connection to express uniform and homogenous connectivity. However, connectivity in some regions becomes increasingly focal during development (Durston et al., 2006), which we captured with a new measure of connectivity that determines the focal maxima of correlations between ROIs. Each approach measures different aspects of connectivity, which can help parse whether connectivity differences in development involve larger brain regions or tend to be more focal within an ROI.

Considering the mixed findings in the literature related to cortical and subcortical functional connectivity, we aimed to determine age related differences in connectivity between pairs of cortical and subcortical regions. In addition, we were interested in determining how functional connectivity patterns differ with age between pairs of subcortical regions. This has not yet been investigated in previous studies. Previous studies examining rs‐fMRI connectivity in typical development included subjects with a broad age range or had small to moderate sample sizes (*n* < 200 in most cases; Cerliani et al., 2015; Fair et al., 2009; Greene et al., 2014; Rubia, 2013; Sato et al., 2015; Solé‐padullés et al., 2015; Supekar et al., 2009). Thus, to reduce heterogeneity, which could contribute to the mixed findings, we used a large sample of 6‐to‐10 year‐old children from a population‐based cohort. By focusing on a narrow age range in a large sample, we aimed to shed new light on brain development within a narrow period of childhood. This age range is particularly interesting because it is a period in which the brain, behavior, and cognition are rapidly maturing (Livy et al., 1997; Mous et al., 2016). This critical phase in development can provide clues into typical brain function, which can then be extended to evaluate mechanisms governing psychopathology.

## 2 MATERIALS AND METHODS

### 2.1 Participants

The participants of this study included a subgroup of children participating in the Generation R Study, which is a large, population‐based prenatal cohort study in Rotterdam, the Netherlands (Jaddoe et al., 2012). Magnetic resonance imaging (MRI) scans were obtained in a total of 1,070 children between 6 and 10 years of age. The protocol for recruitment and study design is described in detail elsewhere (White et al., 2013). General exclusion criteria consisted of severe motor or sensory disorders (deafness or blindness), neurological disorders, moderate to severe head injuries with loss of consciousness, claustrophobia, and contraindications to MRI. Of 1,070 children who visited the research center for an MRI, 964 children underwent an rs‐fMRI scan. Of those children, 227 were screened as having problem behaviors using the Child Behavior Checklist (see description below) and were excluded from the analyses. Furthermore, subjects were excluded due to excessive head motion (*n* = 88), failed registrations (*n* = 21), failed or low quality cortical segmentations (*n* = 126), less than 125 volumes left after data scrubbing (*n* = 5) and an incidental finding (*n* = 1). The final dataset included 411 subjects. Informed consent was obtained from parents, and all procedures were approved by the Medical Ethics Committee of the Erasmus MC, University Medical Center Rotterdam.

### 2.2 Behavioral and IQ assessment

The children were assessed for behavioral and emotional problems using the Child Behavior Checklist (CBCL/1½‐5), which is a questionnaire filled out by their mothers (93%) or fathers (7%; Achenbach & Rescorla, 2000). The CBCL is a 99‐item inventory covering various behaviors reported by parents. It uses a Likert response format (i.e., “not true”, “somewhat true” and “very true”). The CBCL was used to select children without problem behavior to ensure that associations were independent of major behavioral problems. This was accomplished by excluding participants with a score above the clinical cutoff on any syndrome (98th percentile), DSM‐oriented (98th percentile), or broadband scale (91st percentile), according to Dutch norms (Tick, van der Ende, Koot, & Verhulst, 2007). Furthermore, to minimize the potential for residual confounding, the square root of the sum of all items was used to compute a total problem score to be used as a covariate in analyses.

Two subtests from a Dutch nonverbal IQ test (i.e., Snijders‐Oomen Niet‐verbale intelligentie test, revisie [Tellegen, Winkel, Wijnberg‐Williams, & Laros, 2005]) were conducted, as described in Ghassabian et al. (2014). The mosaics subtest assessed spatial visualization abilities. The categories subtest assessed abstract reasoning abilities.

### 2.3 MR‐image acquisition

Magnetic resonance imaging data were acquired on a General Electric MR‐750 3‐Tesla whole‐body scanner (General Electric, Milwaukee, WI) using a standard 8‐channel, receive‐only head coil. A three‐plane localizer was run first and used to position all subsequent scans. Structural T_1_‐weighted images were acquired using a fast spoiled gradient‐recalled echo (FSPGR) sequence (TR = 10.3 ms, TE = 4.2 ms, TI = 350 ms, NEX = 1, flip angle = 16°, matrix = 256 × 256, field of view (FOV) = 230.4 mm, slice thickness = 0.9 mm). Echo planar imaging was used for the rs‐fMRI session with the following parameters: TR = 2,000 ms, TE = 30 ms, flip angle = 85°, matrix = 64 × 64, FOV = 230 mm × 230 mm, slice thickness = 4 mm. In a previous study the number of TRs necessary for functional connectivity analyses was determined, and therefore the first set of acquisitions acquired 250 TRs (acquisition time = 8 min 20 s; White et al., 2014). After it was determined that fewer TRs provided stable networks of higher quality (less motion), the number of TRs was reduced to 160 (acquisition time = 5 min 20; White et al., 2014). Children were instructed to keep their eyes closed and not to think about anything in particular during the rs‐fMRI scan. After the scan session they were asked how the scan went and whether they fell asleep during the scan.

### 2.4 MR‐image processing

#### 2.4.1 Anatomical Image Processing

Predefined ROIs were defined in native space and used as the anatomical regions to quantify time‐series data for brain‐wide connectivity analysis. A total of 34 cortical regions and seven subcortical ROIs were defined in each hemisphere of the brain in native space from T_1_‐weighted images using the FreeSurfer analysis suite (https://surfer.nmr.mgh.harvard.edu; Fischl et al., 2004). Details about the FreeSurfer data processing and quality control in the Generation R Study are described elsewhere (Mous et al., 2014). The FreeSurfer image, including the cortical and subcortical labels were registered to the rs‐fMRI data by applying the transformation matrix resulting from a 12 degree of freedom affine registration of the T_1_‐weighted image to the rs‐fMRI data (Greve & Fischl, 2009). Thus, all time‐series for analyses were extracted from native fMRI space.

#### 2.4.2 Resting‐state image processing

Resting‐state fMRI data were preprocessed using a combination of tools from the Analysis of Functional NeuroImages package (AFNI; Cox, 1996), the Functional MRI of the Brain Software Library (FSL; Jenkinson, Beckmann, Behrens, Woolrich, & Smith, 2012), and in‐house software written in Python version 2.7.3. For the rs‐fMRIs acquired with 250 TRs, only the first 160 volumes were used so that all time courses contained the same amount of information. Preprocessing of the rs‐fMRI began with slice‐timing correction, motion correction, removing the first four volumes, and 0.01 Hz high‐pass temporal filtering. Next, the six motion correction parameters, the mean white matter signal and mean cerebral spinal fluid (CSF) signal were regressed out of each voxel's time course (Fox, Zhang, Snyder, & Raichle, 2009). Finally, data scrubbing was used to further compensate for motion, removing volumes with excessive movement (i.e., greater than 0.5 mm root mean squared relative motion; Power, Barnes, Snyder, Schlaggar, & Petersen, 2012, 2013) since head motion during scanning can amplify developmental differences in connectivity (Power et al., 2012). This effect is significantly reduced after compensating for movement (Di Martino et al., 2014).

Given geometric distortions resulting from susceptibility artifacts, some ROIs were excluded from the analyses. In order to identify affected ROIs, FSL's Brain Extraction Tool (Smith, 2002) was used to create a brain mask from the rs‐fMRI. The proportion of voxels in each ROI that intersected with the brain mask was computed for each subject. Overlap between voxels believed to represent true signal (i.e., within the brain mask) was found to be low in ROIs known to be affected by susceptibility artifacts. ROIs with a mean overlap across subjects of less than 90% were visually inspected and those ROIs with consistently low overlap were excluded from the analyses (entorhinal cortex, frontal pole, inferior temporal gyrus, lateral orbitofrontal cortex, medial orbitofrontal cortex, and temporal pole). In the remaining ROIs, only voxels in the intersection of the ROI and the brain mask were included in the analyses. See Table 1 for a listing of included ROIs.

**Table 1:** Regions used in connectome analysis, grouped by location in the brain

Cluster	Region	Abbreviation
Frontal (Fro)	Caudal anterior cingulate cortex	Cac
	Caudal middle frontal gyrus	Cmf
	Isthmus of cingulate gyrus	ICG
	Paracentral lobule	PCe
	Pars opercularis	POp
	Pars orbitalis	POb
	Pars triangularis	PTr
	Posterior cingulate gyrus	PCi
	Precentral gyrus	PrC
	Rostral anterior cingulate gyrus	RAC
	Rostral middle frontal gyrus	RMF
	Superior frontal gyrus	SFr
Occipital (Occ)	Cuneus	Cun
	Lateral occipital gyrus	LOc
	Lingual gyrus	Lin
	Pericalcarine cortex	Pcc
Parietal (Par)	Inferior parietal lobule	IPa
	Postcentral gyrus	PoC
	Precuneus	Pcn
	Superior parietal lobule	SPa
	Supramarginal gyrus	SMa
Subcortical (Sub)	Accumbens area	Acc
	Amygdala	Amg
	Caudate	CaN
	Hippocampus	Hip
	Pallidum	Pal
	Putamen	Put
	Thalamus	Tha
Temporal (Temp)	Banks of superior temporal sulcus	BSt
	Fusiform gyrus	Fus
	Insula	Ins
	Middle temporal gyrus	MTe
	Parahippocampal gyrus	Phc
	Superior temporal gyrus	STe
	Transverse temporal gyrus	TrT

### 2.5 Brain‐wide connectivity analysis

Brain‐wide connectivity analyses were conducted in rs‐fMRI native space, after the FreeSurfer labels were mapped to the rs‐fMRI data. The labels and preprocessed rs‐fMRI data were used to calculate pairwise region‐to‐region functional connectivity. Before calculating functional connectivity, a 3 × 3 × 3 voxel median spatial filter was applied to the preprocessed rs‐fMRI to increase the signal to noise ratio. Two types of functional connectivity matrices were calculated. First, the connection weight for each pair of ROIs was calculated using a Pearson correlation coefficient of the mean time‐series between all pairs of ROI's (MeanTS). For the second approach, Pearson correlation coefficients were computed between all pairs of voxels within two ROIs, and the pair with the highest Pearson correlation coefficient was selected to represent the connection between those two ROIs. We coin this approach the “Anatomic and Local Peak Activity Correlation Analysis” (ALPACA). The first approach represents connectivity which is homogeneous over a pair of ROIs, whereas the second approach represents the peak connectivity which is localized to focal areas within a pair of ROIs.

For both types of connectivity, only voxels that were part of the fMRI brain mask were considered. This minimized voxels affected by geometric distortions from influencing the connection weight. Prior to statistical analyses, to satisfy normality assumptions for parametric statistics, Pearson correlation coefficients were converted to a normal distribution using the Fisher's *r*‐to‐*z* transformation.

### 2.6 Statistical analysis

Statistical analyses were conducted with the statsmodels (Seabold & Perktold, 2010), scipy (Oliphant, 2007) and numpy (Van Der Walt, Colbert, & Varoquaux, 2011) packages in Python (v2.7). For each connection, two regression models were fitted, one for MeanTS and one for ALPACA. In both cases, age, gender, and the CBCL total problem score were included as independent variables, and main effects were examined for age and gender. The CBCL total problem score was included to account for residual behavioral differences among included children. To control for multiple testing, the number of effective independent tests/connections, *M*_eff_, was computed for both ALPACA and MeanTS according to the method outlined in (Li, Yeung, Cherny, & Sham, 2012). The threshold of significance was determined using the Sidak correction, $\alpha_{\mathrm{corr}}=1-{\left(1-\alpha \right)}^{\left(1/M_{\mathrm{eff}}\right)}$, where *α* = 0.05. We additionally conducted a separate analysis in which interaction between age and gender was tested by adding an interaction term to the model. Multiple testing was controlled using the same thresholds as in the main‐effects model.

### 2.7 Visualization

Connectograms (van Horn et al., 2012) were used to visualize associations of age and gender with functional connectivity. Connectograms are used in brain connectivity analyses to show relationships between ROIs in a circular two‐dimensional representation. ROIs are positioned around the outside of the circle. A given connection is represented by a line between the associated ROIs, where color and thickness are used to indicate specific properties of a connection. In this study, ROIs were grouped by anatomy (see Table 1 for groupings) and by hemisphere. Only connections with significant associations are shown. Red and blue represent positive and negative associations with age or male > female and female > male in the case of gender respectively. Increased color intensity represents increased significance. Connectograms are often easier to interpret than three‐dimensional representations of connectivity in anatomical space (Langen, White, Ikram, Vernooij, & Niessen, 2015).

Worm plots were used to directly compare groups of connections between MeanTS and ALPACA (Langen et al., 2015). Each point represents the association between the variable of interest and a specific connection. Connection‐age association significance is on the *y*‐axis, which is the negative log of the *p*‐value, multiplied by the sign of the association and a scaling factor that is used to ensure that the line representing significance is at the same location for both connectivity types. Connections were ordered along the *x*‐axis according to the anatomical group to which their ROIs belonged (see Table 1 for the list of ROIs belonging to each group). Groups were ordered by their mean associations with ALPACA. Within each group of connections, points were ordered by their association, which produces a worm‐like shape. This allows easy comparison of association strengths and distributions between connectivity types. The ordering was performed separately for each type of connectivity, which means that the order of connections likely differs between connection types. Points that are outside of the dashed lines indicate connections with significant associations after correction for multiple testing.

## 3 RESULTS

**Figure 1:**
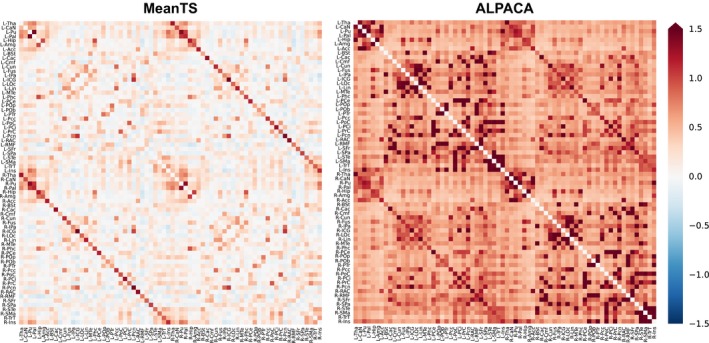
Mean connectomes across subjects for MeanTS and ALPACA. Each element in the matrix represents one connection, where connection weight is the Fisher *r*‐to‐*z* transformation of the correlation between the corresponding regions on the *x*‐ and *y*‐axes

Sample characteristics are reported in Table 2. Mean age was 8 years and 206 subjects were female. The majority of subjects (372 of 411) were right‐handed. Mean connectomes across subjects are shown in Figure 1 for both MeanTS and ALPACA.

**Table 2:** Sample characteristics (*N* = 411)

*General*	
Age at MRI (years)	8.05 ± 0.99
Gender (M/F)	205/206
Non‐verbal IQ	103.77 ± 14.40
Handedness (right/left/unknown)	372/38/1
*Ethnicity*	
Dutch (*n*)	316
Nonwestern (*n*)	68
Other western (*n*)	27
*FMRI motion parameters*	
Average RMS relative (mm)	0.11 ± 0.08

**Figure 2:**
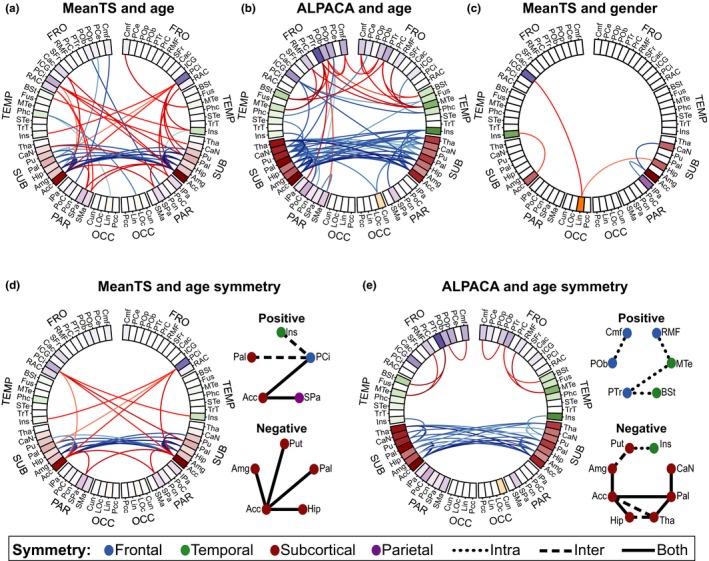
Connectograms (van Horn et al., 2012) showing connections with a significant association of (a) MeanTS with age (b) ALPACA with age and (c) MeanTS with gender. There were no connections with significant associations with gender and ALPACA, therefore the corresponding connectogram is not shown. Brain regions are divided according to location in the brain, including frontal (FRO), temporal (TEMP), subcortical (SUB), parietal (PAR), and occipital (OCC). They are arranged in a circle. Regions from the left hemisphere are on the left side of the diagram. Significant connections between two regions are plotted as red (positive age associations, or male > female) and blue (negative age associations, or female > male) lines, where color intensity indicates relative significance. The opacity of each region indicates the relative number of significant associations that each regions has. The age associations had a great deal of symmetry in both networks, as shown in (d) for MeanTS and (e) for ALPACA. The connectograms in (d) and (e) show the subset of connections that had intrahemispheric (i.e., left‐left and right‐right connections were both significant) and/or interhemispheric (i.e., left‐right and right‐left connections were both significant) symmetry. These connections are also illustrated more abstractly and simply to the right of the connectograms, where regions are represented by circles, connections are represented by lines and the appearance of each line indicates the type of symmetry

Numerous age‐related connections had significant associations that survived correction for multiple testing. These are shown in connectograms in Figure 2 and are summarized in Table 3. Negative associations with age (i.e., weaker connection strength in older children) were dominant, including 24 of 48 (50%) connections in MeanTS and 66 of 84 (79%) in ALPACA. A large proportion of negative associations with age were found in subcortical‐to‐subcortical connections, including 18 of 24 (75%) in MeanTS and 38 of 66 (58%) in ALPACA. Significant age associations with cortical‐to‐subcortical connections were primarily positive in the MeanTS approach (17 of 19 connections, 89%) but negative in all 22 of ALPACA's significant associations with age. This suggests that functional connectivity between subcortical and cortical regions increases homogenously over the entire volume of the involved regions, but decreases focally with age. Positive associations involved all lobes except for the occipital lobes in both approaches and subcortical regions in ALPACA. No significant interactions between age and gender were found for either ALPACA or MeanTS in any of the connections, once corrected for multiple testing. *T*‐tests and Pearson correlations also showed no significant relationship between age and IQ, gender and IQ as well as age and mean displacement. There was a significant Pearson correlation between age and mean displacement (−0.15, *p* < 0.05), however, we adjusted for motion as described in the methods section.

**Table 3:** Location of significant associations

		Hemisphere			Total
		Left	Right	Between	
Age	ALPACA	30	20	34	84
	Positive	8	5	5	18
	Fro/Fro	2	1	1	4
	Fro/Par	2	0	0	2
	Fro/Temp	4	4	4	12
	Negative	22	15	29	66
	Fro/Par	1	0	0	1
	Fro/Sub	3	0	1	4
	Fro/Temp	0	1	3	4
	Occ/Sub	0	2	1	3
	Par/Par	1	0	0	1
	Par/Sub	3	2	0	5
	Sub/Sub	12	6	20	38
	Sub/Temp	2	4	4	10
	MeanTS	13	12	23	48
	Positive	5	7	12	24
	Fro/Par	2	1	0	3
	Fro/Sub	2	3	5	10
	Fro/Temp	0	2	2	4
	Par/Sub	1	1	4	6
	Sub/Temp	0	0	1	1
	Negative	8	5	11	24
	Fro/Par	1	0	1	2
	Fro/Sub	1	0	0	1
	Occ/Temp	1	0	1	2
	Sub/Sub	5	4	9	18
	Sub/Temp	0	1	0	1
Gender	MeanTS	2	2	1	5
	Positive	2	0	1	3
	Fro/Occ	1	0	0	1
	Occ/Sub	0	0	1	1
	Sub/Temp	1	0	0	1
	Negative	0	2	0	2
	Par/Sub	0	1	0	1
	Sub/Sub	0	1	0	1

The age connectograms were relatively symmetric, suggesting that both homogeneous and focal age‐related differences occur similarly in both hemispheres in the brain. Specific connections with symmetric age associations are shown in Figure 2d,e, where symmetry is intrahemispheric (i.e., both ROI_A,left_‐to‐ROI_B,left_ and ROI_A,right_‐to‐ROI_B,right_ are significant), interhemispheric (i.e., both ROI_A,left_‐to‐ROI_B,right_ and ROI_A,right_‐to‐ROI_B,left_ are significant), or both. The nucleus accumbens played a central role in symmetry in negative associations, which were primarily in connections between subcortical regions in both network approaches. Positive symmetry involved frontal, temporal, parietal, and subcortical regions.

**Figure 3:**
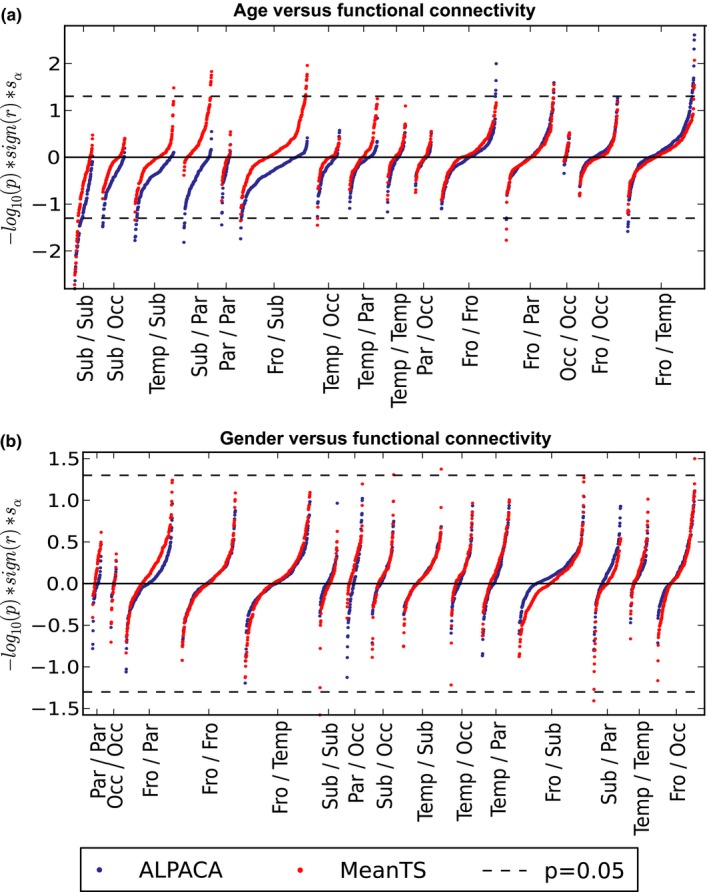
Worm plots (Langen et al., 2015) of association of functional measures with age and gender. Connections are split into groups based on the location of the associated regions, including frontal (Fro), temporal (Temp), subcortical (Sub), parietal (Par), and occipital (Occ). Connections within each group are ordered by association strength, producing worm‐like shapes. Order of groups on the *x*‐axis is ordered by mean association strength in ALPACA. On the *y*‐axis is the negative log of the *p*‐value, multiplied by the sign of the test, multiplied by a scaling factor. Each point outside of the dotted lines represents a significant association of age or gender with a specific connection: Worm plots (Langen et al., 2015) of association of functional measures with age and gender. Connections are split into groups based on the location of the associated regions, including frontal (Fro), temporal (Temp), subcortical (Sub), parietal (Par), and occipital (Occ). Connections within each group are ordered by association strength, producing worm‐like shapes. Order of groups on the *x*‐axis is ordered by mean association strength in ALPACA. On the *y*‐axis is the negative log of the *p*‐value, multiplied by the sign of the test, multiplied by a scaling factor. Each point outside of the dotted lines represents a significant association of age or gender with a specific connection

Figure 3 shows the distribution of connection weights grouped by lobe using a worm plot (Langen et al., 2015). Most subcortical/parietal and subcortical/frontal connection associations with age were positive in MeanTS but negative in ALPACA. In other words, in this group of edges homogenous functional connectivity increases with age, however, there are focal areas where functional connectivity decreases with age. There were few connectivity differences between gender using both the ALPACA and MeanTS approaches. MeanTS had a total of five significant associations with gender, including three in which connectivity in males was stronger than in females (left isthmus cingulate/left lingual, left accumbens/left insula, and left lingual/right hippocampus) and two where females had greater connectivity than males (right accumbens/right caudate and right accumbens/right inferior parietal cortex). ALPACA did not identify any significant associations after correction for multiple testing. This suggests that gender‐related differences in connectivity are homogeneous across the involved ROIs rather than focal.

## 4 DISCUSSION

In this study, we examined age‐ and gender differences in functional connectivity by applying two different, but complementary approaches to measure functional connectivity. Both connectivity approaches revealed both common and different patterns of connectivity in relation to age, and relatively similar patterns of connectivity between boys and girls. Significant associations between connectivity and age revealed a concentration of negative associations with age between pairs of subcortical regions and positive associations between pairs of cortical regions. The age associations generally displayed left‐right symmetry. Additionally, when connections were grouped anatomically, group‐wise shifts in associations with age were found. The two different connectivity indices were overall highly consistent; however, there were a number of connections where they diverged, suggesting that in a subset of connections, functional connectivity changes with age either homogeneously or focally over the involved ROIs, but not both.

### 4.1 Connectivity increases in the cortex and decreases in the subcortex with age

Both methods derived several cortico‐cortical connections that were positively associated with age. This is consistent with a recent study that found that cortico‐cortical connectivity increases during development in children from seven to 18 years of age (Solé‐padullés et al., 2015). Our findings expand upon this finding by demonstrating that age‐related increases in connectivity are present in a narrow age‐range in young children while utilizing two different methods for deriving connectivity indices. This increase in connectivity parallels an increase in volume of the frontal, temporal, and parietal lobes, which has been reported to occur between the 6–10 years of age (Lenroot & Giedd, 2006). Thus, the increased volume, which may be a result of synaptogenesis and arborization, may also result in increasing cross‐talk between brain regions. Previous studies have found that functional connectivity increases with age in long‐range connections and decreases in short‐range connections (Fair et al., 2009; Rubia, 2013). This is partially consistent with our observations, since many of the identified significant positive associations were in connections between regions in different lobes and/or hemispheres, and were thus medium to long‐range connections. We did, however, find a small number of both long‐range connections that decreased with age and short‐range connections that increased with age. Thus, maturation of brain connectivity may be region dependent, with many long‐range connections increasing with age, whereas some show decreases. While the regions with positive associations differed between the two connectivity types, both support the notion of generally increasingly distributed networks with age. Our observations are particularly interesting because we focused on a narrow age range, whereas many previous studies focused on relatively large age ranges (Fair et al., 2009; Rubia, 2013). It is remarkable that such striking connectivity differences with age can be observed even within a narrow age range in school‐age children. This is likely a result of the rapid neurodevelopment that occurs during this age range. In addition, since movement during MRI scanning shows strong age‐related differences, with children having greater movement than adolescents and adults, the narrow age range used in our study provides greater similarity in movement parameters compared to studies with larger age ranges (Fair et al., 2009; Rubia, 2013) and thus is less biased by age‐related movement artifacts.

Age associations with connections between cortical and subcortical regions differed between network approaches. MeanTS had a mix of positive and negative associations, while ALPACA had exclusively negative associations with age, adding new insight into the nature of previously observed changes in connectivity with age. The negative associations in ALPACA suggest that focal connectivity between cortical and subcortical regions decreases with age, which is consistent with studies reporting negative associations with age in connections between subcortical and cortical regions in typical development (Cerliani et al., 2015; Greene et al., 2014; Sato et al., 2015; Supekar et al., 2009). However, (Solé‐padullés et al., 2015) found primarily positive as well as some negative age associations between cortico‐subcortical connections, and (Sato et al., 2015) found that the thalamus had both positive and negative association with age in development. Our results in MeanTS, which is an expression of functional connectivity that is homogenous over the involved regions, also support the presence of subcortical‐to‐cortical connection associations in both directions. Under the rubric of specific functional brain networks or cortico‐subcortical feedback loops associated with neurodevelopment (i.e., the cortico‐cerebellar‐thalamic‐cortical circuit [CCTCC]; Andreasen & Pierson, 2008; Ullsperger, Danielmeier, & Jocham, 2014), the presence of both positive and negative associations between cortical and subcortical regions may be expected. Maturing feedback loops involving similar functions would show increasing connectivity with age, whereas those involved in different functions would show less age‐related functional connectivity. Significant differences of cortico‐subcortical functional connectivity with age are also parallel to previously observed increases in size of the frontal, temporal and parietal lobes as well as some subcortical regions (Lenroot & Giedd, 2006).

While there is a wealth of developmental studies examining cortical‐to‐cortical connections, and to a lesser extent subcortical‐to‐cortical connections, there is a gap in the literature regarding age‐related differences in the connectivity between different subcortical structures. In this study, we found that all significant associations of connectivity between subcortical regions with age were negative for both network types. Our findings between subcortical structures may reflect networks transforming from local to distributed during development, as was shown by (Fair et al., 2009). However, their study focused on cortical and cerebellar regions, and did not report on subcortical/subcortical connectivity.

Structural MRI studies of subcortical structures examined how volumes of subcortical regions change over time (Lenroot & Giedd, 2006). These changes include an inverted U‐shaped pattern in the volume of the caudate with peaks at 7.5 and 10.0 years of age in females and males, respectively; an increase in hippocampal size in males only and an increase in the size of the amygdala in girls only. The amygdala, hippocampus and caudate were involved in subcortical connections with negative associations with age, which was true for both networks for the amygdala and hippocampus, and only for ALPACA in the caudate. As these regions have been shown to increase in volume during childhood and subsequently decrease during adolescence (Sowell, Thompson, & Toga, 2004), their communications with other subcortical regions likely also change during development. It is thus possible that in the presence of later maturing cortical structures in young children (i.e., prefrontal cortex; Lenroot & Giedd, 2006; Mills, Goddings, Clasen, Giedd, & Blakemore, 2014), subcortical structures rely on within‐system connectivity. As the cortex matures and its connections to the subcortex strengthen (Cummings, 1993), this previous subcortical reliance on highly integrative connectivity may be relaxed. Such an imbalance in timing of development has been previously proposed for cortical/limbic connectivity (Casey, Jones, & Hare, 2008; Heller, Cohen, Dreyfuss, & Casey, 2016). Given the importance of various subcortical structures and their cortical connections with different psychiatric disorders (e.g., Cortico‐cerebellar‐thalamic‐cortical loop in Schizophrenia, caudate motor in ADHD, thalamus/basal ganglia/primary sensory networks; Cerliani et al., 2015), having a better understanding of differences within and between cortical and subcortical regions is a crucial foundation for future efforts studying connectivity differences related to psychopathology.

An interesting finding in this study was inter‐ and intrahemispheric symmetry in age associations. Symmetry in the negative associations in both network types was primarily between subcortical regions with the nucleus accumbens playing a central role, whereas positive symmetry involved frontal, temporal, parietal, and subcortical regions. This suggests that many bilateral connections within and between hemispheres are developing simultaneously. The fact that many subcortical connections with the accumbens area had negative associations with age in both network types might be related to development of the reward center of the brain. The accumbens has been linked to risk‐taking behavior in adolescents (Galvan et al., 2006), but previous studies have not directly investigated the development of subcortical connection to the amygdala in children. Our results suggest that activity is increasingly directed by cortical regions rather than subcortical regions. Asymmetry in brain connectivity has previously been observed in lateralization studies (Agcaoglu, Miller, Mayer, Hugdahl, & Calhoun, 2015; Di, Kim, Chen, & Biswal, 2014; Holland et al., 2007). Adolescent and adult brains are highly lateral across several resting state networks, with several brain regions showing a decrease in lateralization with age (Agcaoglu et al., 2015). In children, language networks become increasingly left‐lateralized throughout development (Groen, Whitehouse, Badcock, & Bishop, 2012; Holland et al., 2007), whereas visuospatial networks become right‐lateralized (Groen et al., 2012). Although lateralization of the brain may be related to asymmetric association of functional connectivity with age, this relationship has not been studied directly, nor can it be definitively assumed. Lateralization can increase even if the association with age is significant on both sides of the brain. While symmetry in functional connectivity has been widely studied, the symmetry of *associations with* functional connectivity have not. Examination of association symmetry could be informative in future studies. For example, individual deviations from the symmetry pattern found in typical development could be used as a marker of psychopathology.

### 4.2 Sexual dimorphism

Five connections had significant associations surviving correction of multiple testing of MeanTS with gender. ALPACA did not have any associations with gender. Together these results suggest that gender‐related differences in functional connectivity are likely more uniform across the involved regions, rather than being localized to spatially focal peaks. These results could alternately suggest that MeanTS is a more robust measure of sexual dimorphism. Previous studies of gender‐related differences in resting‐state functional connectivity are sparse in this age range. A recent study did not find any gender differences in the age range of 7–12 (Solé‐padullés et al., 2015). Additionally, diffusion tensor MRI study in children aged six to ten found no significant gender‐related differences in measures of white matter integrity (Muftuler et al., 2012). Both studies support our observation of few connectivity differences between gender in this age range.

The lack of observed gender differences in functional connectivity during development in both our study and previous studies are surprising given that studies of structural connectivity have found gender differences in relation to cognition and/or intelligence in children and adolescents. Several previous studies have found gender differences in structural connectivity (Hänggi et al., 2010; Schmithorst, 2009; Simmonds, Hallquist, Asato, & Luna, 2014), however, a recent DTI study in the current cohort did not show gender differences (Muetzel et al., 2015). Gender differences have also previously been observed in neuroanatomical studies. For example, longitudinal structural MRI studies have shown gender differences in grey matter volume in the frontal, parietal, and temporal lobes, as well as in the caudate, amygdale, and hippocampus from childhood throughout adolescence (Lenroot & Giedd, 2006). In this study, all of these regions with the exception of the amygdala had connections with significant associations with gender. Given that previous work present conflicting views on gender differences in connectivity and related grey matter volumes, and since our study found a small number of connections with gender differences in only one of the two functional networks studied, it seems that gender differences in functional connectivity are subtle and limited in typically developing children in this age range. Measureable gender differences in the brain may emerge or become unmasked with development, with differences between boys and girls may become more apparent during adolescence and young adulthood.

### 4.3 Defining functional connectivity by peak activation versus over an entire region

As described above, both network types were generally in agreement with each other and with the existing literature. In some specific connections, some differences were apparent across method with respect to associations in specific connections. In the case of such differences, this suggests that the nature of the development of functional connectivity is not the same for all regions. For example, MeanTS did not have significant associations with age in fronto‐frontal connections, whereas ALPACA's positive associations with age were exclusively found in fronto‐frontal, fronto‐temporal, and fronto‐parietal connections. This is in line with findings of an earlier study that suggested that cortical connections become increasingly focal with age (Durston et al., 2006). This is in contrast with age associations with the posterior cingulate, which were positive in MeanTS but not ALPACA. This suggests that developmental changes in posterior cingulate connectivity are distributed across the entire structure rather than localized in a focal region. Previous studies have shown that connectivity in the default mode network changes during development, including connections involving the posterior cingulate (Fair et al., 2008; Supekar et al., 2010).

Increasingly diffuse connectivity with age was also found in cortical‐to‐subcortical connections, which were primarily positive in MeanTS but exclusively negative in ALPACA. This thus suggests a focal to diffuse trajectory with age. Such a trajectory in subcortical‐to‐subcortical connections was not found since their age associations were exclusively negative in both network types.

It is interesting to consider the differences between the two network types in the context of the underlying neuronal architecture. If connectivity with grey matter is more diffuse, with connecting neurons covering a more extensive surface of an ROI, then a more diffuse representation, such as MeanTS, would better capture changes in functional connectivity (e.g., a “shared pathway”). On the other hand, if axonal pathways between two regions start and end in focal gray matter locations, then a focal representation of functional connectivity, such as ALPACA, may target critical regions of connectivity.

There are additional factors that must be kept in mind interpreting results involving ALPACA. For example, ALPACA's focal approach may be more flexible in identifying the location of activation because it does not average over entire regions, which can blur the signal. This may be advantageous in relation to both structural and functional variability because it may not always be sensible to assume the same spatial activation patterns across individuals. On the other hand, ALPACA does not guarantee that the activation detected across individuals corresponds to the same focal connection. For example, it may be that a large region has more than one focal peak in connectivity. ALPACA may thus choose one peak for some subjects and another for others, in which case comparison across individuals would not involve the same connection. Additionally, in some cases, weaker functional connectivity has been related to some forms of psychopathology (e.g., autism [Ha, Sohn, Kim, Sim, & Cheon, 2015] and depression [Hermesdorf et al., 2015]). In this situation, finding the local maximum may not be desirable in the context of better explaining the neurobiological underpinnings of psychopathology or identifying novel biomarkers because the local maxima may not necessarily reflect the reduced connectivity across the involved regions. Given the benefits and drawbacks and the underlying assumptions of each network type, using both ALPACA and MeanTS simultaneously in future studies may result in greater insights into different aspects of functional connectivity and make inferences of whether a given connection has a diffuse or focal connectivity pattern.

### 4.4 Strengths and limitations

While most studies on developmental functional connectivity focus on broad age ranges with moderate sample sizes (Rubia, 2013), many of which used task‐based fMRI rather than resting state fMRI, our study focused on a narrow age range and benefited from increased statistical power due to the large cohort. The children included in this study were sampled from a population‐based cohort and were representative of the general population, which helped to mitigate the common issue of selection bias of children with higher than average IQ or greater socioeconomic status. An additional strength of this study is that, by keeping our analysis in native space, our results were not influenced by intersubject registration, which has frequently been used in previous studies and has been shown to blur cortical areas (Fischl, Sereno, Tootell, & Dale, 1999; White et al., 2001). This study also effectively used “brain‐wide” visualizations to display large amounts of connectomic information, namely in the connectograms and worm plots. In addition, we present both novel findings as well as replication of observations from earlier studies, the latter being important in neuroscience, which is a field plagued by many underpowered studies that do not replicate (Nichols et al., 2017; Open Science Collaboration, 2015).

As previously mentioned, we used a FreeSurfer anatomical segmentation to define our regions of interest. Anatomical segmentations have also been used in several previous studies (Cammoun et al., 2012; Fornito, Yoon, Zalesky, Bullmore, & Carter, 2011; Tadayonnejad, Yang, Kumar, & Ajilore, 2014). This approach benefits from a subject‐specific segmentation in native space, which does not require intersubject registrations. Studies that include intersubject registrations are vulnerable to misregistration (Di Martino et al., 2014). This approach may, however, fall short in the event that ROIs are not functionally specific or homogeneous. Choice of segmentation can affect the results of connectomics studies (de Reus & van den Heuvel, 2013). Functionally defined ROIs can be obtained using fMRI. Existing methods define regions to be either nonoverlapping (Blumensath et al., 2013; Shen, Tokoglu, Papademetris, & Constable, 2013; Yeo et al., 2011) or overlapping (Beckmann, 2012; van den Heuvel & Hulshoff Pol, 2010; Smith et al., 2012, 2013). For example, (Yeo et al., 2011) used functional MRI to define a cortical segmentation that maximized functional specialization within regions across subjects. The borders of the resulting functional ROIs were significantly different from the anatomically defined ROIs used in this study. Thus, a functional ROI may intersect with several anatomical ROIs. Additionally, an anatomical ROI could be composed of several functionally distinct regions or may be part of a larger functional region. Because MeanTS averages signals over ROIs, some of which are quite large, imprecise boundaries would likely be less of a problem than they would be for ALPACA. In the event that a given region contains several functionally distinct subregions, ALPACA runs the risk of choosing different subregions across subjects for the same connection. However, in the case of large anatomical regions, where only a part of the ROI is active, the MeanTS approach would average over the entire region, which would not reflect activity in the active region. This may result in underestimated functional connectivity between regions. The ALPACA approach would circumvent this by choosing the highest activation and the number of voxels involved in calculating the correlation coefficient are always the same.

In order to reduce the possibility of spurious correlations we applied a median filter. This approach runs the risk that connectivity between highly focal voxels may be diminished via the spatial smoothing. Thus, we chose to smooth only using the 28 voxels surrounding the voxel of interest. Given a voxel dimension of 3.4 mm × 3.4 mm × 4.0 mm, the total size of the smoothed voxel including the median filter is 1,248 mm^3^, which is a reasonably large smoothing kernel for native space and should help reduce chance findings due to noise spikes within the data. We have shown previously that not only structural variability, but also functional variability contributes to differences in the anatomic locations of fMRI signals (White et al., 2001), and thus specific voxels may not be spurious correlations, but rather the higher intensity may be the result of a true underlying focal neural signal that differs spatially between participants.

We did not evaluate the variability in the spatial location of the ALPACA‐derived peaks. Larger brain regions, such as many of the FreeSurfer‐defined cortical regions, may have multiple peaks where the relative magnitude of peaks may vary between individuals, which could be interpreted as greater functional heterogeneity. The subcortical regions, being smaller than many of the cortical regions, are less likely to have multiple peaks and thus this likely explains the similarity in findings between the ALPACA and MeanTS approaches. Future studies should assess the heterogeneity in the number and locations of peaks within FreeSurfer regions within the context of development.

This study measured alertness by asking subjects to report whether they fell asleep in the scanner. While none of the children reported falling asleep, we did not measure EEG activity and thus it is possible that some of the children may have slept during the scans. This could have an effect on the results of this study.

Functional connectivity studies, and particularly those involving pediatric populations, are frequently impacted by motion artifacts, which can erroneously increase long‐range connectivity and decrease short‐range connectivity (Fornito, Bullmore, & Zalesky, 2017; Di Martino et al., 2014; Power et al., 2014) Given that younger children tend to move more than older children, this can have an impact on developmental studies. In this study, we corrected for motion using the “scrubbing” method (Power et al., 2012, 2013), where corrupted volumes are removed. While this method significantly reduces the effect of motion (Power et al., 2014), it is but one of many strategies (Di Martino et al., 2014). Among the drawbacks of the scrubbing method are the loss of data within subjects, and the unequal degrees of freedom across subjects (Power et al., 2014).

Another issue relevant to connectome‐wide association studies is multiple testing correction. This study calculated the “number of effective tests” for each network type based on the covariance in the data, and used this number to adjust the significance threshold. Some of the differences in associations between the two networks investigated in this study could be simply due to the threshold chosen for each network. This is one of many similar methods commonly used in genetics studies to approximate permutation testing (Sham & Purcell, 2014). Permutation testing has been used previously in connectomics (Ingalhalikar et al., 2014), but remains a computationally expensive method of multiple testing correction. Another option is to reduce the number of tests by using measures such as the network‐based statistic (Zalesky, Fornito, & Bullmore, 2010), or to consider graph theoretical measures that produce node‐ or graph‐level values (Kaiser, 2011; Rubinov & Sporns, 2010). This approach has been used in several studies (Betzel et al., 2014; Crossley et al., 2014; Fornito et al., 2011; Fornito, Zalesky, Pantelis, & Bullmore, 2012; Zhou, Gennatas, Kramer, Miller, & Seeley, 2012), however, it fundamentally shifts the research focus from identification of relevant connections to the interpretation of measures that often do not have a known relation to neuro‐biology (Smith, 2012). Lastly, this study included individuals from the general population, rather than solely recruiting “typically developing” children from the community. We utilized a common behavioral and emotional problem inventory to exclude children with high levels of behavior problems to maximize comparability of these data with the existing literature. While most behavioral and emotional problems are robustly measured by this parent‐report instrument, the children themselves may arguably be better informants for some types of problem behavior (e.g., internalizing vs. externalizing problems). However, even with some misclassification of problem behavior, the population‐based nature of the present sample is highly useful in that it greatly increases the generalizability of findings across all individuals of the population, rather than only the “typically developing” individuals.

## 5 CONCLUSION

The current study provides both replication and novel findings for age‐related maturation of intrinsic connectivity. Replication of findings is noteworthy given our large sample size and narrow age range, coupled with critique regarding less than optimal reproducibility and replication in the field of neuroimaging. Cortico‐cortico connectivity was found to increase with age, while connectivity between subcortical regions decreased with age. Some cortico‐cortical connections became increasingly focal with age, whereas other cortico‐cortical and most cortico‐subcortical connections became more diffuse with age. Additionally, we demonstrate the utility of native‐space analyses of connectivity and offer examples of how the data can be efficiently and intuitively displayed. Future studies should explore using different anatomical or functional parcellations to determine to what extent the connectivity patterns are influenced by ROI boundaries.
